# Traditional Chinese Medicine Use among Patients with Psoriasis in Taiwan: A Nationwide Population-Based Study

**DOI:** 10.1155/2016/3164105

**Published:** 2016-10-16

**Authors:** Shu-Wen Weng, Bor-Chyuan Chen, Yu-Chiao Wang, Chun-Kai Liu, Mao-Feng Sun, Ching-Mao Chang, Jaung-Geng Lin, Hung-Rong Yen

**Affiliations:** ^1^Graduate Institute of Chinese Medicine, College of Chinese Medicine, China Medical University, Taichung, Taiwan; ^2^Department of Chinese Medicine, Taichung Hospital, Ministry of Health and Welfare, Taichung, Taiwan; ^3^Department of Chinese Medicine, Dalin Tzu Chi Hospital, Buddhist Tzu Chi Medical Foundation, Chiayi, Taiwan; ^4^Management Office for Health Data, China Medical University Hospital, Taichung, Taiwan; ^5^Department of Chinese Medicine, China Medical University Hospital, Taichung, Taiwan; ^6^School of Chinese Medicine, College of Chinese Medicine, China Medical University, Taichung, Taiwan; ^7^Research Center for Chinese Medicine & Acupuncture, China Medical University, Taichung, Taiwan; ^8^Center for Traditional Medicine, Taipei Veterans General Hospital, Taipei, Taiwan; ^9^Graduate Institute of Clinical Medicine and Graduate Institute of Traditional Chinese Medicine, College of Medicine, Chang Gung University, Taoyuan, Taiwan; ^10^Research Center for Traditional Chinese Medicine, Department of Medical Research, China Medical University Hospital, Taichung, Taiwan

## Abstract

Traditional Chinese medicine (TCM) has long been used for patients with psoriasis. This study aimed to investigate TCM usage in patients with psoriasis. We analyzed a cohort of one million individuals representing the 23 million enrollees randomly selected from the National Health Insurance Research Database in Taiwan. We identified 28,510 patients newly diagnosed with psoriasis between 2000 and 2010. Among them, 20,084 (70.4%) patients were TCM users. Patients who were female, younger, white-collar workers and lived in urbanized area tended to be TCM users. The median interval between the initial diagnosis of psoriasis to the first TCM consultation was 12 months. More than half (*N* = 11,609; 57.8%) of the TCM users received only Chinese herbal medicine. Win-qing-yin and Bai-xian-pi were the most commonly prescribed Chinese herbal formula and single herb, respectively. The core prescription pattern comprised Mu-dan-pi, Wen-qing-yin, Zi-cao, Bai-xian-pi, and Di-fu-zi. Patients preferred TCM than Western medicine consultations when they had metabolic syndrome, hepatitis, rheumatoid arthritis, alopecia areata, Crohn's disease, cancer, depression, fatty liver, chronic airway obstruction, sleep disorder, and allergic rhinitis. In conclusion, TCM use is popular among patients with psoriasis in Taiwan. Future clinical trials to investigate its efficacy are warranted.

## 1. Introduction

Psoriasis is a common chronic immune-mediated inflammation disease. The prevalence rates of psoriasis ranged from 0.7% to 2.9% in Europe, 0.7 to 2.6% in the United States [1], and 0.235% in Taiwan [2]. The burden of psoriasis was estimated as $35.2 billion in 2013 in the United States [3], while the estimated annual total cost for psoriasis was $53.8 million in Taiwan [4]. The cost of long-term therapy and social economic burden of psoriasis have posed a significant impact on healthcare system.

Current treatment for psoriasis is not fully satisfactory. Several types of dermatological treatment are available, ranging from corticosteroids, vitamin D analogs, and phototherapy for mild-moderate psoriasis to retinoids, methotrexate, cyclosporine, apremilast, and biologic immune modifying agents for severe psoriasis [5]. However, many patients are concerned about the side effects of these treatments. Skin atrophy may be induced by long-term use of topical corticosteroids [6]. Common adverse events of topical steroid and vitamin D agent may be partial local irritation and skin pain [7]. Apremilast may cause nausea, upper respiratory tract infection, and diarrhea [8]. Phototherapy may increase the risk of skin cancer [9]. Methotrexate was associated with the increased risk of liver injury [10]. Oral cyclosporin may have the risk of kidney toxicity [11]. A web-based survey study showed that patients with psoriasis were moderately satisfied with their treatment [12].

Traditional Chinese medicine (TCM), which includes acupuncture and moxibustion, Chinese traumatology, and Chinese herbal medicine (CHM), has been integrated as an important part of healthcare in Taiwan. It has been commonly used for dermatitis [13], gastrointestinal disease [14], rheumatoid arthritis [15], diabetic mellitus [16, 17], gynecological disorder [18], and cancer [19, 20] patients. Previous studies showed that CHM plus acitrerin had add-on effect [21] and Chinese herbal bath combined with phototherapy was superior to phototherapy alone [22] for psoriasis. Topical application of Lindioil, extract of Qing-dai (Indigo Naturalis;* Baphicacanthus cusia* (Nees) Bremek,* Polygonum tinctorium* Aiton,* Isatis indigotica* Fortune ex Lindl.) in oil, was effective for treating nail psoriasis [23] and Chinese herbal ointment that contains Qing-dai was effective for plaque-type psoriasis [24].

In the United States, 2.0% of patients with psoriasis received complementary and alternative medicine (CAM) therapy [25]. Despite the growing interests in utilizing CAM, there remains a critical knowledge gap on the ethnopharmacological analysis of the TCM prescription patterns for psoriasis patients.

In Taiwan, the compulsory National Health Insurance (NHI) system was launched in 1995 and the NHI program started to reimburse TCM service in 1996. As of 2015, The NHI program covered 99.6% of Taiwanese population [26]. To investigate the prescription patterns of CHM for psoriasis patients, we took advantage of the National Health Insurance Research Database (NHIRD), which contains registration files and claims data for reimbursement. We analyzed a cohort of one million beneficiaries from the NHIRD from 2000 to 2010. This study is important to delineate the TCM utilization patterns among patients with psoriasis. It could be regarded as a consensus of TCM formulas/herbs for further pharmacological investigation or clinical trials.

## 2. Material and Methods

### 2.1. Data Source

This study used datasets from the NHIRD (http://nhird.nhri.org.tw/en/) in Taiwan as our previous reports [14, 18]. The NHI program in Taiwan reimbursed TCM services (CHM, acupuncture/moxibustion, and Chinese traumatology therapy) provided by licensed TCM doctors. The NHI database consists of registration files and original claim data for reimbursement. The large-scale computerized data derived from the NHI program were maintained by the National Health Research Institutes, Taiwan, and provided to scientists in Taiwan for research purposes. All registration data in the NHIRD consist of demographic characteristics, diagnosis, clinical visits, hospitalization, procedures, prescriptions, and the medical costs for reimbursement [27]. The diagnostic codes were in the International Classification of Diseases, Ninth Revision, Clinical Modification (ICD-9-CM) formats.

### 2.2. Study Population and Variables

**Figure 1:**
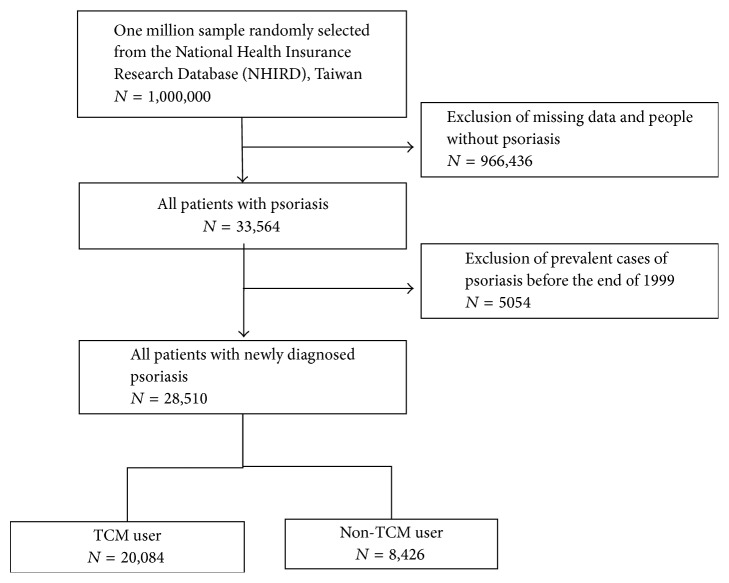
Flow recruitment chart of subjects from the one million random samples obtained from the National Health Insurance Research Database (NHIRD) from 2000 to 2010 in Taiwan.

The flow chart of enrolling psoriasis patients was shown in Figure 1. A one million random sample from the NHIRD was selected for this study. All the patients (*n* = 28,510) with newly diagnosed psoriasis (ICD-9-CM code: 696) between January 2000 and December 2010 were included in this study and then followed up until the end of 2011. After a confirmed diagnosis with psoriasis, those consulted with TCM doctors were grouped as TCM users (*n* = 20,084) and the others as non-TCM users (*n* = 8426). We investigated the demographic characteristics of TCM users and non-TCM users, including sex, age, occupation, urbanization, and the time between being diagnosed of psoriasis and TCM treatment. We analyzed the incidence rate ratio of diseases between TCM and non-TCM users. We also analyzed ten most common herbal formulas and single herbs prescribed by TCM doctors for psoriasis patients. Herbal formulas were listed in pin-yin name and English name. Single herbs were listed in pin-yin name, Latin name, and botanical plant name. TCM indications of the Chinese herbal formulas and single herbs were based on TCM theory [28, 29]. Full botanical names comply with the* International Plant Names List* (IPNI; http://www.ipni.org/) and* The Plant List* (http://www.theplantlist.org/) [30].

### 2.3. Core Patterns of Chinese Herbal Medicine

The core pattern of CHM used in treating psoriasis patients was identified with an open-sourced freeware NodeXL (http://nodexl.codeplex.com/), and all the selected two drugs combinations were applied in this network analysis. The line width, ranging from 1 to 5 in the network figure, was defined by counts of connections between a CHM and coprescribed CHM, and thicker widths of line connections indicated a significant prescription pattern [31]. The network analysis manifested the core pattern of the top 50 two-drug combinations in this survey.

### 2.4. Ethical Considerations

All of the information that could be used to identify individuals or care providers were deidentified and encrypted before release. It is not possible to identify any individuals or care providers at any level in this database. This study was approved by the Research Ethics Committee of China Medical University and Hospital (CMUH104-REC2-115).

### 2.5. Statistical Analysis

We used the SAS software, version 9.4 (SAS Institute Inc., Cary, NC, USA), to analyze the datasets retrieved from the NHIRD. Descriptive statistics was applied to determine the demographic characteristics, treatment modalities, and the frequency of prescribed herbal formulas and single herbs. The diagnoses were coded according to the International Classification of Diseases, Ninth Revision, Clinical Modification (ICD-9-CM) codes. To present the overall structure of the study groups, we showed the mean and standard deviation (SD) for age and number and percentage for sex and comorbidity. To assess the distribution difference between TCM and non-TCM users, the t-test was used for continuous variable (age) and chi-square test was used for category variables (sex, occupation, urbanization, and major disease categories/diagnosis). The crude and adjusted prevalence rate ratio (PRR) and 95% confidence intervals (CIs) of those particular diseases for the TCM users compared with non-TCM users were estimated using Poisson regression models. A *p* value of <0.05 was considered as statistically significant.

## 3. Results

There were 28,510 patients who were newly diagnosed with psoriasis (Table 1). Among them, 20,084 (70.4%) patients ever used TCM outpatient services. The majority (54.9%) of the TCM users were female. The mean age of TCM users was younger than that of non-TCM users (30.0 versus 34.5 years old). The majority (59.2%) of the TCM users were white-collar workers. Most of the TCM users resided in urbanized areas. The average time between onset of psoriasis and the first visit to a TCM clinic was 12.0 months.

Regarding the treatment modalities given to patients with psoriasis, 11,609 (57.8%) patients received only CHM, while 214 (1.1%) patients were treated by acupuncture or Chinese traumatology only and 8,261 (41.1%) patients received the combination of both treatments. More than half of the patients (*n* = 11,037; 55.0%) visited TCM clinics for more than 6 times per year (Table 2).

To investigate the prescription patterns of the Chinese herbal remedies, we conducted a comprehensive analysis and identified ten most commonly prescribed Chinese herbal formulas (Table 3) and single herbs (Table 4), respectively. The most frequently prescribed Chinese herbal formula was Win-qing-yin (Warm Clearing Beverage). Regarding the single herbs for the treatment of psoriasis, Bai-xian-pi (Cortex Dictamni;* Dictamnus dasycarpus* Turcz.) was the most commonly prescribed single herb.

**Figure 2:**
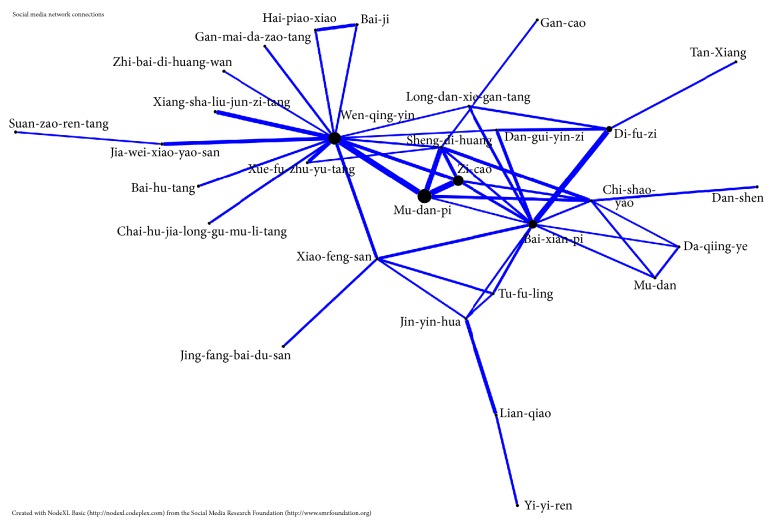
The top 50 combinations of herbal formulas and single herbs for psoriasis patients were analyzed through open-sourced freeware NodeXL. The core prescription pattern was Mu-dan-pi, Wen-qing-yin, Zi-cao, Bai-xian-pi, and Di-fu-zi.

To further investigate the core prescription patterns, we conducted the network analysis. We found that Mu-dan-pi (Cortex Moutan;* Paeonia suffruticosa* Andrews), Win-qing-yin (Warm Clearing Beverage), Zi-cao (Radix Lithospermi;* Lithospermum erythrorhizon* Siebold & Zucc.;* Arnebia euchroma* (Royle) I.M.Johnst.;* Arnebia guttata* Bunge), Bai-xian-pi (Cortex Dictamni;* Dictamnus dasycarpus* Turcz.), and Di-fu-zi (Fructus Kochiae;* Kochia scoparia* (L.) Schrad.) composed the core prescription patterns of CHM to treat psoriasis patients (Figure 2).

Comparing the disease prevalence rate ratio of TCM and non-TCM group, we found that psoriasis patients with certain disease tended to consult TCM service more than Western medicine consultation (Table 5). Specifically, psoriasis patients preferred to visit TCM doctors when they had metabolic syndrome, hepatitis, rheumatoid arthritis, alopecia areata, Crohn's disease, cancer, depression, fatty liver, chronic airway obstruction, sleep disorder, and allergic rhinitis.

## 4. Discussion

This nationwide population-based study analyzed a cohort of one million beneficiaries from the NHIRD to investigate the TCM usage among patients with psoriasis. We found that approximately 70.4% of the patients with psoriasis visited TCM clinics. Psoriasis patients with female gender, a younger age, residency in urbanized area, and white collar had a tendency to consult TCM service. The most commonly adopted TCM treatment is CHM. We also identified the ten most common prescribed Chinese herbal formulas and single herbs for the treatment of psoriasis. Wen-qing-yin and Bai-xian-pi were the most commonly used TCM herbal formula and single herb, respectively. Overall, our study provided valuable information and added value to the existing knowledge regarding TCM treatment for patients with psoriasis.

In our study, women, compared with men, were more likely to use TCM. We found that the highest TCM utilization rate was young people. Several surveys also showed similar findings [14, 32]; women and younger people had a higher usage of TCM. There was also a tendency for TCM users to reside in higher urbanized area. This may be because highly urbanized areas have a high density of TCM doctors in Taiwan [32].

Patients with psoriasis utilized healthcare services significantly more often than those without psoriasis [33], and we found that half of patients with psoriasis used TCM. In this study, patients visited TCM services on an average of 12.0 months after initial diagnosis of psoriasis. Probably most patients usually used Western medicine as the first choice initially. If patients were unsatisfied with conventional therapy, recurrent symptoms, or high costs of biological agents, they might further seek adjunctive TCM consultation [34, 35].

In accordance with other studies in atopic dermatitis or urticaria [13, 36], CHM is the most common treatment approaches. We found that only very few patients received acupuncture therapy. Regarding the visiting frequency, a majority of the patients used TCM service for more than 6 times, which might be due to the confidence in symptoms relief or effectiveness perceived by users. However, for those who received both treatment modalities, they tended to use TCM services more frequently. It is possible that those patients who chose a combination of CHM and acupuncture or traumatology therapy had complicated situations that required more frequent treatment.

Psoriasis is not a disease merely affecting skin. We found that patients with psoriasis were associated with a high degree of incidence rate ratio for visiting TCM clinics when they suffered from various medical comorbidities. Previous investigations were consistent with our findings that psoriasis patients had a broad spectrum of disease category in their clinical visits [2, 37].

According to the theory of traditional medicine, psoriasis is further identified by different patterns, such as blood heat, heat toxin, damp heat, blood stasis, and blood dryness. Our network analysis in Figure 2 included the top 50 combinations of herbal formulas and herbs for psoriasis patients. According to the theory of TCM theory, this core pattern can clear heat, cool the blood, nourish the blood, dispel wind, and dry dampness.

Wen-qing-yin was the most frequently prescribed formula for psoriasis. It was originally documented in an ancient literature as “Wan Bing Hui Chun.” This formula is composed of Si-wu-tang (Four Agents Decoction) and Huang-lian-jie-du-tang (Coptis Toxin-Resolving Decoction). Together in this formula, they can clear heat, transform dampness, and nourish the blood based on the TCM theory. In a previous report, Wen-qing-yin could inhibit the induction phase of various kinds of delayed type hypersensitivity and local graft-versus-host reactions [38]. The second commonly prescribed herbal formula is Xiao-feng-san (Wind-Dispersing Powder). It has long been used to treat skin problems in clinical practice such as atopic dermatitis [13] or urticaria [36]. In a clinical trial conducted in our institution, Xiao-feng-san was found to improve refractory atopic dermatitis symptom and with no side effects [39]. In basic studies, Xiao-feng-san as an antiallergic drug was reported to inhibit IgE dependent histamine release from the cultured mast cells [40]. Xiao-feng-san might also correct the Th1/Th2 balance by preventing the increase in interleukin-4 mRNA expression and the decrease in interferon-gamma mRNA expression to inhibit dermatitis [41]. Long-dan-xie-gen-tang (Gentian Liver-Draining Decoction) is the third most common formula. In clinical practice, Long-dan-xie-gen-tang is commonly prescribed to subjects with chronic hepatitis [42], atopic dermatitis [13], and insomnia [43] in Taiwan. In TCM viewpoint, it can drain fire and clear damp heat. Besides the symptoms of skin, patients with psoriasis often suffered from sleep problems [2, 44]. Whether Long-dan-xie-gen-tang has direct effect on skin or indirect efficacy on comorbidity, such as insomnia, deserves further investigation.

Bai-xian-pi (Cortex Dictamni;* Dictamnus dasycarpus* Turcz.) was the most commonly prescribed single herb. It can clear heat and dry dampness, dispel wind, and resolve toxin. Among the prescribed single herbs, seven of top ten single herbs belonged to the category of “clearing heat.” Tropical treatment of methanol extract of* Dictamnus dasycarpus* Turcz. root bark on dermatitis mice could effectively inhibit skin thickness, hyperplasia, and edema [45]. The anti-inflammatory effects of* Dictamnus dasycarpus* Turcz. may reduce the level of *β*-hexosaminidase and histamine release [46]. Mu-dan-pi (Cortex Moutan;* Paeonia suffruticosa* Andrews) was used to clear heat and cool the blood in TCM. Previous study found that the water extract of Moutan Cortex not only inhibited *β*-hexosaminidase and tumor necrosis factor-*α* release in IgE-mediated DNP-BSA-stimulated RBL-2H3 cells but also improved the compound 48/80-induced allergic reactions in a mouse model [47]. Zi-cao (Radix Lithospermi;* Lithospermum erythrorhizon* Siebold & Zucc.;* Arnebia euchroma* (Royle) I.M.Johnst.;* Arnebia guttata* Bunge) has been used to clear blood heat and has shown an anti-inflammatory effect in experimental studies [48]. Its ingredient, shikonin, was found to suppress IL-17 signaling in keratinocytes [49]. Di-fu-zi (Fructus Kochiae;* Kochia scoparia* (L.) Schrad.) has been used to clear heat and dry dampness. It was reported to ameliorate dermatitis via inhibition of the production of proinflammatory cytokines [50]. Sheng-di-huang (Radix Rehmanniae;* Rehmannia glutinosa* (Gaertn.) DC.) polysaccharides extract was found to increase skin glutathione, superoxide dismutase, catalase, and glutathione peroxidase activities and decrease skin malondialdehyde level in ultraviolet B ray treated mice, suggesting that it may be useful for skin diseases [51].

Taken together with the classification and principles of TCM formulas and single herbs, our findings corresponded with the viewpoint of TCM, which believes that the occurrence of psoriasis symptoms is related to the blood heat, blood stasis, and blood dryness. However, it has to be pointed out that future validation on their efficacy and safety is necessary.

The strength of this study at least included the following aspects: First, all residents of Taiwan can access the NHI system with low cost and convenience, and thus the accessibility of healthcare, either Western or Chinese medicine, is high. Second, all patients with psoriasis were included in this study and this nationwide population-based study comprehensively included all the prescriptions for psoriasis patients. These TCM formulas and single herbs may provide some thought in the exploration of better treatment options.

Some caveats in this study merit comments. First, this study did not include topical Chinese herbal ointments or herbal bath, which were not the forms of TCM reimbursed by the NHI program. The NHI program only covers TCM prescriptions manufactured by GMP-certified pharmaceutical companies in Taiwan. Future study to explore the topical herbal products is necessary. Second, the severity of psoriasis and efficacy of CHM were not available in this database. Judging from the fact that 70.4% of the patients with psoriasis used TCM service, it is necessary to conduct clinical trials to evaluate the efficacy and safety of these prescriptions.

## 5. Conclusion

This study is the first large-scale survey to analyze TCM utilization patterns among patients with psoriasis in Taiwan. Patients with psoriasis often sought help from TCM treatment. Wen-qing-yin was the most frequently prescribed Chinese herbal formula, while Bai-xian-pi was the most common single herb. Future clinical trials and pharmacological investigations could be developed based on the findings of this study.

## Figures and Tables

**Table 1:** Demographic characteristics of TCM and non-TCM users among patients with psoriasis from 2000 to 2010 in Taiwan.

Variable	Non-TCM users		TCM users		*p* value
	*N*	(%)	*N*	(%)	
Number of cases	8426	29.6	20084	70.4	
Sex^†^					<0.0001
Female	3457	41.0	11035	54.9	
Male	4969	59.0	9049	45.1	
Age^†^, y					<0.0001
<25	3560	42.3	9601	47.8	
25–35	1275	15.1	3681	18.3	
35–65	2451	29.1	5554	27.7	
≧65	1140	13.5	1248	6.2	
Mean (SD)^#^	34.5 (22.7)		30.0 (18.7)		<0.0001
Occupation^†^					<0.0001
White collar^$^	4716	56.0	11892	59.2	
Blue collar^*※*^	2475	29.4	5805	28.9	
Others^‡^	1235	14.7	2387	11.9	
Urbanization^†^					0.0090
1 (highest)	2487	29.5	5992	29.8	
2	2467	29.3	6007	29.9	
3	1514	18.0	3773	18.8	
4+ (lowest)	1958	23.2	4312	21.5	
Interval between the diagnosis of psoriasis and the first visit to a TCM clinic, months, median (IQR)			12.0 (28.4)		

^†^Chi-square test; ^#^Student's *t*-test.

^$^White collar: civil services, institution workers, enterprise, business, and industrial administration personnel. ^*※*^Blue collar: farmers, fishermen, vendors, and industrial laborers. ^‡^Others: retired, unemployed and low-income populations.

**Table 2:** Frequency distribution of TCM clinic visits and treatment modalities among TCM users from 2000 to 2010 in Taiwan.

Number of TCM visits	Only Chinese herbal medicine *N* = 11609 (57.8%)	Only Acupuncture or traumatology *N* = 214 (1.1%)	Combination of both treatments *N* = 8261 (41.1%)	Total *N* = 20084 (100%)
1–3	4753 (40.9)	201 (93.9)	924 (11.2)	5878 (29.3)
4–6	1903 (16.4)	10 (4.7)	1256 (15.2)	3169 (15.7)
>6	4953 (42.7)	3 (1.4)	6081 (73.6)	11037 (55.0)

**Table 3:** Ten most common herbal formulas prescribed for the treatment of patients with psoriasis from 2000 to 2010 in Taiwan.

Herbal formula		Ingredients of herbal formula			
Pin-yin name	English name	Pin-yin name (Chinese material medica name; botanical name)	Therapeutic actions and indications based on TCM theory	Number	Average daily dose (g)
Wen-qing-yin	Warm Clearing Beverage	Dang-gui (Radix Angelicae Sinensis; *Angelica sinensis *(Oliv.) Diels), Chuan-xiong (Rhizoma Chuanxiong; *Ligusticum chuanxiong *S.H.Qiu, Y.Q.Zeng, K.Y.Pan, Y.C.Tang & J.M.Xu), Bai-shao-yao (Radix Paeoniae Alba; *Paeonia lactiflora *Pall.), Shou-di-huang (Radix Rehmanniae Preparate; *Rehmannia glutinosa *(Gaertn.) DC.), Huang-qin (Radix Scutellariae; *Scutellaria baicalensis *Georgi), Huang-bai (Cortex Phellodendri; *Phellodendron amurense *Rupr.), Zhi-zi (Fructus Gardeniae; *Gardenia jasminoides* J.Ellis), Huang-lian (Rhizoma Coptidis; *Coptis chinensis *Franch.; *Coptis deltoidea *C.Y.Cheng & P.K.Hsiao; *Coptis teeta *Wall.)	Clear heat, transform dampness, and nourish the blood	1450	33.5

Xiao-feng-san	Wind-Dispersing Powder	Jing-jie (Herba Schizonepetae; *Schizonepeta tenuifolia *(Benth.) Briq.), Fang-feng (Radix Saposhnikoviae; *Saposhnikovia divaricata *(Turcz.) Schischk.), Chan-tui (Periostracum Cicadae; *Cryptotympana pustulata *Fabricius), Ren-shen (Radix Ginseng; *Panax ginseng *C.A.Mey.), Gan-cao (Radix Glycyrrhizae; *Glycyrrhiza uralensis *Fisch.; *Glycyrrhiza inflata *Batalin; *Glycyrrhiza glabra *L.), Chuan-xiong (Rhizoma Chuanxiong; *Ligusticum chuanxiong *S.H.Qiu, Y.Q.Zeng, K.Y.Pan, Y.C.Tang & J.M.Xu), Qiang-huo (Rhizoma et Radix Notopterygii;* Notopterygium incisum *K.C.Ting ex H.T.Chang), Jiang-can (Batryticatus Bombyx; *Bombyx mori *Linnaeus), Hou-po (Cortex Magnoliae Officinalis; *Magnolia officinalis *Rehder & E.H.Wilson; *Magnolia officinalis* var. biloba Rehder & E.H.Wilson), Huo-xiang (Herba Pogostemonis; *Agastache rugosa *(Fisch. & C.A.Mey.) Kuntze), Fu-ling (Poria;* Poria cocos *(Schw.) Wolf), Chen-pi (Pericarpium Citri Reticulate; *Citrus reticulata *Blanco)	Course wind and discharge heat	763	25.5

Long-dan-xie-gan-tang	Gentian Liver-Draining Decoction	Long-dan-cao (Radix Gentianae; *Gentiana scabra *Bunge; *Gentiana triflora *Pall.; *Gentiana manshurica *Kitag.), Zhi-zi (Fructus Gardeniae; *Gardenia jasminoides* J.Ellis), Huang-qin (Radix Scutellariae; *Scutellaria baicalensis *Georgi), Chai-hu (Radix Bupleuri; *Bupleurum chinense *DC.), Sheng-di-huang (Radix Rehmanniae; *Rehmannia glutinosa *(Gaertn.) DC.), Ze-xie (Rhizoma Alismatis; *Alisma plantago-aquatica *L.), Dang-gui (Radix Angelicae Sinensis; *Angelica sinensis *(Oliv.) Diels), Che-qian-zi (Semen Plantaginis; *Plantago asiatica *L.; *Plantago depressa *Willd.), Chuan-mu-tong (Caulis Clematidis Armandii; *Clematis armandii *Franch.), Gan-cao (Radix Glycyrrhizae; *Glycyrrhiza uralensis *Fisch.; *Glycyrrhiza inflata *Batalin; *Glycyrrhiza glabra *L.)	Drain liver fire and clear damp heat	517	9.99

Jia-wei-xiao-yao-san	Supplemented Free Wanderer Powder	Dang-gui (Radix Angelicae Sinensis; *Angelica sinensis *(Oliv.) Diels), Fu-ling (Poria;* Poria cocos *(Schw.) Wolf), Zhi-zi (Fructus Gardeniae; *Gardenia jasminoides* J.Ellis), Bo-he (Herba Menthae; *Mentha haplocalyx *Briq.), Bai-shao-yao (Radix Paeoniae Alba; *Paeonia lactiflora *Pall.), Chai-hu (Radix Bupleuri; *Bupleurum chinense *DC.), Gan-cao (Radix Glycyrrhizae; *Glycyrrhiza uralensis *Fisch.; *Glycyrrhiza inflata *Batalin; *Glycyrrhiza glabra *L.), Bai-zhu (Rhizoma Atractylodis Macrocephalae; *Atractylis macrocephala *(Koidz.) Hand.-Mazz.), Mu-dan-pi (Cortex Moutan; *Paeonia suffruticosa* Andrews), Wei-jiang (Rhizoma Praeparatum Zingiberis; *Zingiber officinale *Roscoe)	Rectify Qi and nourish blood	461	20.0

Dang-gui-yin-zi	Chinese Angelica Drink	Dang-gui (Radix Angelicae Sinensis; *Angelica sinensis *(Oliv.) Diels), Chuan-xiong (Rhizoma Chuanxiong; *Ligusticum chuanxiong *S.H.Qiu, Y.Q.Zeng, K.Y.Pan, Y.C.Tang & J.M.Xu), Bai-shao-yao (Radix Paeoniae Alba; *Paeonia lactiflora *Pall.), Sheng-di-huang (Radix Rehmanniae; *Rehmannia glutinosa *(Gaertn.) DC.), Bai-ji-li (Fructus Tribulus; *Tribulus terrestris *L.), Fang-feng (Radix Saposhnikoviae; *Saposhnikovia divaricata *(Turcz.) Schischk.), Jing-jie (Herba Schizonepetae; *Schizonepeta tenuifolia *(Benth.) Briq.), He-shou-wu (Radix Polygoni Multiflori; *Polygonum multiflorum *Thunb.), Huang-qi (Radix Astragali; *Astragalus membranaceus *(Fisch.) Bunge), Gan-cao (Radix Glycyrrhizae; *Glycyrrhiza inflata *Batalin; *Glycyrrhiza glabra *L.), Sheng-jiang (Rhizoma Zingiberis Recens;* Zingiber officinale *Roscoe)	Nourish the blood, moisten dryness, dispel wind, and relieve itching	435	65.4

Xue-fu-zhu-yu-tang	House of Blood Stasis-Expelling Decoction	Dang-gui (Radix Angelicae Sinensis; *Angelica sinensis *(Oliv.) Diels), Sheng-di-huang (Radix Rehmanniae; *Rehmannia glutinosa *(Gaertn.) DC.), Tao-ren (Semen Persicae; *Prunus persica *(L.) Batsch; *Prunus davidiana *(CarriŠre) Franch.), Hong-hua (Flos Carthami; *Carthamus tinctorius* L.), Zhi-ke (Fructus Aurantii; *Citrus aurantium *L.), Chi-shao-yao (Radix Rubra Paeoniae; *Paeonia lactiflora *Pall.), Chai-hu (Radix Bupleuri; *Bupleurum chinense *DC.), Gan-cao (Radix Glycyrrhizae; *Glycyrrhiza inflata *Batalin; *Glycyrrhiza glabra *L.), Jie-geng (Radix Platycodonis; *Platycodon grandiflorus *(Jacq.) A.DC.), Chuan-xiong (Rhizoma Chuanxiong; *Ligusticum chuanxiong *S.H.Qiu, Y.Q.Zeng, K.Y.Pan, Y.C.Tang & J.M.Xu), Niu-xi (Radix Cyathulae; *Achyranthes bidentata *Blume; *Cyathula officinalis *K.C.Kuan)	Quicken the blood, transform stasis, move qi, and relieve pain	430	2.97

Zhi-bai-di-huang-wan	Anemarrhena, Phellodendron, and Rehmannia Pill	Shou-di-huang (Radix Rehmanniae Praeparate; *Rehmannia glutinosa *(Gaertn.) DC.); Shan-zhu-yu (Fructus Corni; *Cornus officinalis *Siebold & Zucc.), Shan-yao (Rhizoma Dioscoreae; *Dioscorea opposita *Thunb.), Fu-ling (Poria;* Poria cocos *(Schw.) Wolf), Mu-dan-pi (Cortex Moutan; *Paeonia suffruticosa* Andrews), Ze-xie (Rhizoma Alismatis; *Alisma plantago-aquatica *L.), Zhi-mu (Rhizoma Anemarrhenae; *Anemarrhena asphodeloides *Bunge), Huang-bai (Cortex Phellodendri; *Phellodendron amurense *Rupr.)	Enrich yin and downbear fire	414	7.85

Huang-lian-jie-du-tang	Coptis Toxin-Resolving Decoction	Huang-qin (Radix Scutellariae; *Scutellaria baicalensis *Georgi), Huang-bai (Cortex Phellodendri; *Phellodendron amurense *Rupr.), Zhi-zi (Fructus Gardeniae; *Gardenia jasminoides* J.Ellis), Huang-lian (Rhizoma Coptidis; *Coptis chinensis *Franch.; *Coptis deltoidea *C.Y.Cheng & P.K.Hsiao; *Coptis teeta *Wall.)	Drain fire and resolve toxin	347	8.95

Xiang-sha-liu-jun-zi-tang	Costusroot and Amomum Six Gentlemen Decoction	Mu-xiang (Radix Aucklandiae; *Aucklandia lappa *DC.;* Vladimiria souliei* (Franch.) Ling), Sha-ren (Fructus Amomi; *Amomum villosum* Lour.; *Amomum longiligulare* T.L.Wu; *Amomum xanthioides *Wall. ex Baker), Chen-pi (Pericarpium Citri Reticulate; *Citrus reticulata *Blanco), Ban-xia (Rhizoma Pinelliae; *Pinellia ternata *(Thunb.) Makino), Ren-shen (Radix Ginseng; *Panax ginseng *C.A.Mey.), Bai-zhu (Rhizoma Atractylodis Macrocephalae; *Atractylis macrocephala *(Koidz.) Hand.-Mazz.), Fu-ling (Poria;* Poria cocos *(Schw.) Wolf), Gan-cao (Radix Glycyrrhizae; *Glycyrrhiza inflata *Batalin; *Glycyrrhiza glabra *L.), Sheng-jiang (Rhizoma Zingiberis Recens;* Zingiber officinale *Roscoe), Da-zao (Fructus Jujubae; *Ziziphus jujuba *Mill.)	Boost qi, fortify spleen, move qi, and reduce phlegm	284	5.32

Jing-fang-bai-du-san	Schizonepeta and Saposhnikovia Toxin-Vanquishing Powder	Jing-jie (Herba Schizonepetae; *Schizonepeta tenuifolia *(Benth.) Briq.), Fang-feng (Radix Saposhnikoviae; *Saposhnikovia divaricata *(Turcz.) Schischk.), Chai-hu (Radix Bupleuri; *Bupleurum chinense *DC.), Fu-ling (Poria;* Poria cocos *(Schw.) Wolf), Jie-geng (Radix Platycodonis; *Platycodon grandiflorus *(Jacq.) A.DC.), Chuan-xiong (Rhizoma Chuanxiong; *Ligusticum chuanxiong *S.H.Qiu, Y.Q.Zeng, K.Y.Pan, Y.C.Tang & J.M.Xu), Du-huo (Radix Angelicae Pubescentis; *Angelica pubescens *Maxim.), Zhi-ke (Fructus Aurantii; *Citrus aurantium *L.), Gan-cao (Radix Glycyrrhizae; *Glycyrrhiza uralensis *Fisch.; *Glycyrrhiza inflata *Batalin; *Glycyrrhiza glabra *L.), Sheng-jiang (Rhizoma Zingiberis Recens;* Zingiber officinale *Roscoe)	Promote sweating, resolve the exterior, disperse wind, and dispel dampness	211	10.5

**Table 4:** Top ten most common single herbs prescribed for the treatment of patients with psoriasis from 2000 to 2010 in Taiwan.

Single herb					
Pin-yin name	Chinese materia medica name	Botanical name	Therapeutic actions and indications based on TCM theory	Number	Average daily dose (g)
Bai-xian-pi	Cortex Dictamni	*Dictamnus dasycarpus* Turcz.	Clearing heat, drying dampness, dispelling wind, and resolving toxin	783	1.52
Mu-dan-pi	Cortex Moutan	*Paeonia suffruticosa* Andrews	Clearing heat, cooling the blood, quickening the blood, and dispersing stasis	721	3.88
Sheng-di-huang	Radix Rehmanniae	*Rehmannia glutinosa *(Gaertn.) DC.	Clearing heat, cooling the blood, nourishing yin, and engendering liquid	602	5.98
Tu-fu-ling	Rhizoma Smilacis Glabrae	*Smilax glabra* Roxb.	Resolving toxin and drying dampness	502	7.28
Dan-shen	Radix Salviae Miltiorrhizae	*Salvia miltiorrhiza *Bunge	Clearing and quickening the blood and regulating menstruation	479	1.30
Zi-cao	Radix Lithospermi	*Lithospermum erythrorhizon *Siebold & Zucc.; *Arnebia euchroma *(Royle) I.M.Johnst.; *Arnebia guttata *Bunge	Clearing and quickening the blood, resolving toxin, and outthrusting papules	469	1.38
Di-fu-zi	Fructus Kochiae	*Kochia scoparia* (L.) Schrad.	Clearing heat, drying dampness, and relieving itching	440	8.44
Lian-qiao	Fructus Forsythiae	*Forsythia suspensa *(Thunb.) Vahl	Clearing heat, resolving toxin, coursing wind, and dispersing heat	440	23.5
Chi-shao-yao	Radix Rubra Paeoniae	*Paeonia lactiflora *Pall.	Clearing heat, cooling the blood, dispersing stasis, and relieving pain	402	1.31
Jin-yin-hua	Flos Lonicerae	*Lonicera japonica *Thunb.	Clearing heat, resolving toxin, coursing wind, and dispersing heat	392	16.8

**Table 5:** Prevalence rate ratio of diseases between non-TCM and TCM users.

	Non-TCM *N* = 8426		TCM *N* = 20084		Compared to non-TCM	
	*n*	%	*n*	%	Crude PRR	Adjusted PRR^†^
Metabolic syndrome						
Hypertension	1817	21.6	3173	15.8	0.73 (0.70–0.77)^*∗∗∗*^	1.18 (1.11–1.26)^*∗∗∗*^
Diabetes	1000	11.9	1727	8.6	0.72 (0.69–0.76)^*∗∗∗*^	1.14 (1.05–1.24)^*∗∗*^
Hyperlipidemia	1221	14.5	2666	13.3	0.92 (0.87–0.96)^*∗∗∗*^	1.29 (1.20–1.39)^*∗∗∗*^
Heart disease	1379	16.4	2707	13.5	0.82 (0.78–0.86)^*∗∗∗*^	1.20 (1.11–1.28)^*∗∗∗*^
Infections						
Tuberculosis	153	1.82	198	0.99	0.54 (0.51–0.58)^*∗∗∗*^	0.94 (0.74–1.19)
Hepatitis B	376	4.46	1193	5.94	1.33 (1.25–1.42)^*∗∗∗*^	1.46 (1.29–1.65)^*∗∗∗*^
Hepatitis C	184	2.18	435	2.17	0.99 (0.92–1.06)	1.39 (1.16–1.68)^*∗∗∗*^
Auto immune disorder						
Rheumatoid arthritis	15	0.18	48	0.24	1.34 (1.22–1.48)^*∗∗∗*^	1.93 (1.03–3.60)^*∗*^
Systemic lupus erythematosus	16	0.19	25	0.12	0.66 (0.60–0.71)^*∗∗∗*^	0.59 (0.29–1.17)
Vitiligo	40	0.47	123	0.61	1.29 (1.18–1.41)^*∗∗∗*^	1.11 (0.77–1.62)
Pemphigoid	16	0.19	16	0.08	0.42 (0.39–0.46)^*∗∗∗*^	0.86 (0.39–1.89)
Pemphigus	15	0.18	14	0.07	0.39 (0.36–0.43)^*∗∗∗*^	0.58 (0.26–1.32)
Alopecia areata	88	1.04	297	1.48	1.42 (1.31–1.54)^*∗∗∗*^	1.36 (1.06–1.75)^*∗*^
Crohn's disease	449	5.33	1610	8.02	1.50 (1.41–1.60)^*∗∗∗*^	1.32 (1.18–1.47)^*∗∗∗*^
Cancer	329	3.90	597	2.97	0.76 (0.71–0.81)^*∗∗∗*^	1.24 (1.07–1.44)^*∗∗*^
Others						
Depression	379	4.50	1200	5.97	1.33 (1.24–1.42)^*∗∗∗*^	1.41 (1.25–1.60)^*∗∗∗*^
Hyperthyroidism	157	1.86	400	1.99	1.07 (0.99–1.15)	0.99 (0.81–1.21)
Hypothyroidism	49	0.58	153	0.76	1.31 (1.20–1.43)^*∗∗∗*^	1.20 (0.85–1.70)
Multiple sclerosis	5	0.06	15	0.07	1.26 (1.14–1.39)^*∗∗∗*^	1.02 (0.35–2.99)
Fatty liver	144	1.71	331	1.65	0.96 (0.90–1.04)	1.30 (1.05–1.61)^*∗∗*^
Chronic airways obstruction	348	4.13	452	2.25	0.54 (0.51–0.58)^*∗∗∗*^	1.19 (1.02–1.39)^*∗*^
Sleep disorder	1046	12.4	4407	21.9	1.77 (1.68–1.86)^*∗∗∗*^	1.77 (1.65–1.90)^*∗∗∗*^
Asthma	1008	12.0	2303	11.5	0.96 (0.91–1.01)	0.98 (0.91–1.06)
Allergic rhinitis	1956	23.2	6687	33.3	1.43 (1.37–1.50)^*∗∗∗*^	1.26 (1.20–1.33)^*∗∗∗*^

PRR: prevalence rate ratio. ^†^Model adjusted for age, sex, occupation, urbanization, and number of outpatient visits for traditional Chinese medicine. ^*∗*^ *p* ≤ 0.05; ^*∗∗*^ *p* ≤ 0.01; ^*∗∗∗*^ *p* ≤ 0.001.
